# Ca^2+^-activated KCa3.1 potassium channels contribute to the slow afterhyperpolarization in L5 neocortical pyramidal neurons

**DOI:** 10.1038/s41598-020-71415-x

**Published:** 2020-09-02

**Authors:** M. V. Roshchin, V. N. Ierusalimsky, P. M. Balaban, E. S. Nikitin

**Affiliations:** Institute of Higher Nervous Activity and Neurophysiology, Russian Academy of Sciences, 5a Butlerova str., Moscow, 117485 Russia

**Keywords:** Intrinsic excitability, Cellular neuroscience, Ion channels in the nervous system

## Abstract

Layer 5 neocortical pyramidal neurons are known to display slow Ca^2+^-dependent afterhyperpolarization (sAHP) after bursts of spikes, which is similar to the sAHP in CA1 hippocampal cells. However, the mechanisms of sAHP in the neocortex remain poorly understood. Here, we identified the Ca^2+^-gated potassium KCa3.1 channels as contributors to sAHP in ER81-positive neocortical pyramidal neurons. Moreover, our experiments strongly suggest that the relationship between sAHP and KCa3.1 channels in a feedback mechanism underlies the adaptation of the spiking frequency of layer 5 pyramidal neurons. We demonstrated the relationship between KCa3.1 channels and sAHP using several parallel methods: electrophysiology, pharmacology, immunohistochemistry, and photoactivatable probes. Our experiments demonstrated that ER81 immunofluorescence in layer 5 co-localized with KCa3.1 immunofluorescence in the soma. Targeted Ca^2+^ uncaging confirmed two major features of KCa3.1 channels: preferential somatodendritic localization and Ca^2+^-driven gating. In addition, both the sAHP and the slow Ca^2+^-induced hyperpolarizing current were sensitive to TRAM-34, a selective blocker of KCa3.1 channels.

## Introduction

The neocortex is the largest part of the cortex (~ 90% of the entire cortex in humans), and plays a crucial role in higher functions of the brain such as interpretation of sensory information, formation and storage of long-term memory, language usage, and control of voluntary movements. Pyramidal neurons play a key role in all cortical activities and constitute ~ 80% of all neocortical neurons. Neocortical layer 5 is of great interest and importance because the pyramidal neurons of layer 5 integrate information from local neurons and project output signals to distant cortical and subcortical areas^1,2^. Previous studies demonstrated that differences in spike frequency adaptation are important in distinguishing functionally distinct subpopulations of L5 pyramidal cells^3–5^. Based on this criterion, pyramidal cells are classified into two broad classes: regular spiking neurons with weak adaptation and regular spiking with strong adaptation^6^.

**Figure 1:**
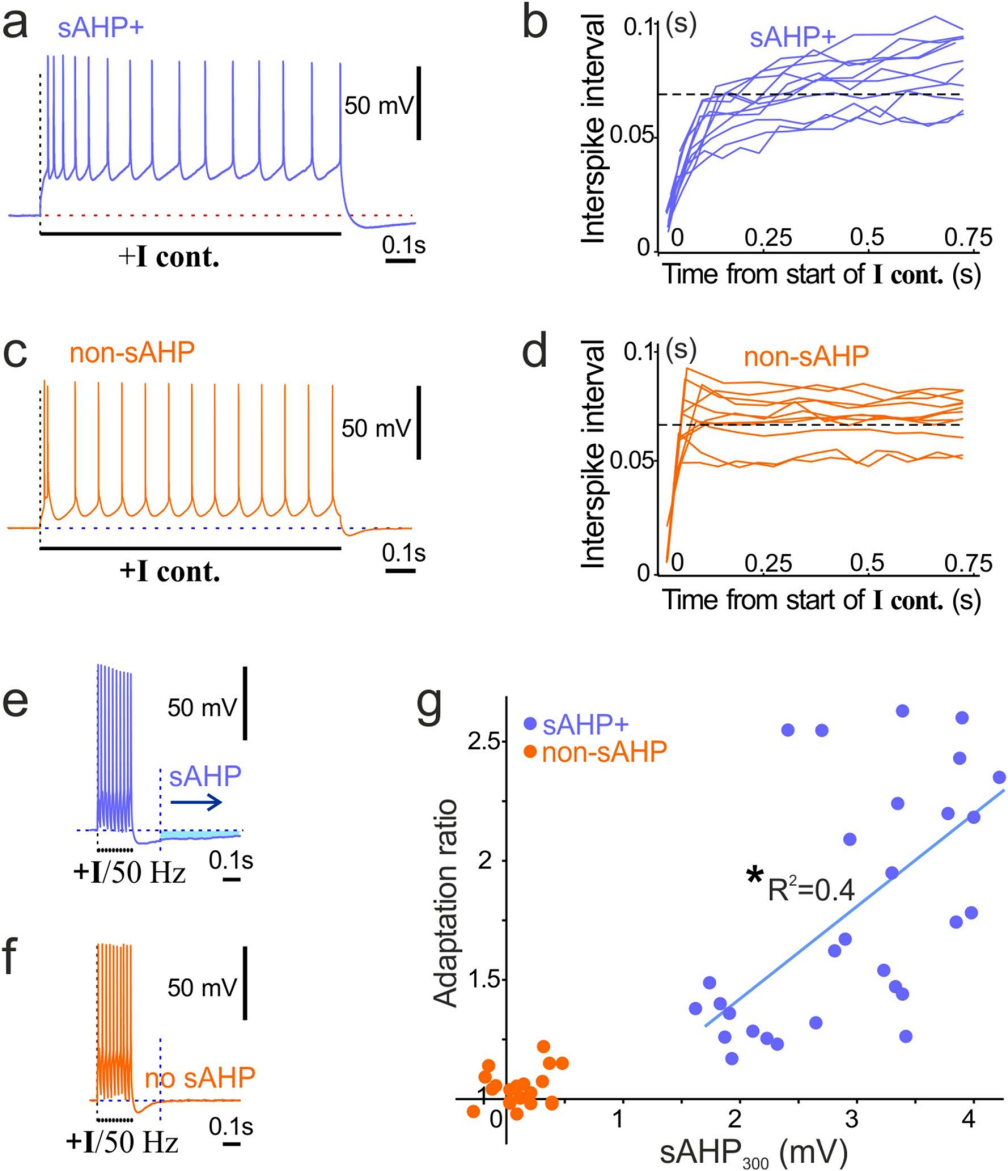
Comparison between sAHP+ cells and non-sAHP cells. (**a**) Example of the adaptation pattern of sAHP+ cells evoked by a continuous current step and (**b**) the corresponding interspike intervals plotted against time from the start of the current step (n = 13 cells). (**c**) Example of the adaptation pattern of non-sAHP cells evoked by a continuous current step and (**d**) the corresponding interspike intervals plotted against time (n = 13 cells). (**e**) Example of afterhyperpolarization (AHP, sky blue area and arrow) evoked by a 50-Hz train of 10 current pulses in a sAHP+ cell. Each pulse evoked a single action potential. (**f**) Example of afterhyperpolarization evoked by a 50-Hz train of 10 current pulses in a non-sAHP cell (no afterhyperpolarization after the dotted bar: 300 ms). (**g**) Summary diagram of adaptation ratios plotted against amplitudes of sAHP, measured at the time point marked with a dotted line in “(**e**)” and “(**f**)” (sAHP_300_). Cells with sAHP (blue circles) and without it (non-sAHP, orange circles) are shown. The blue line illustrate a linear regression of sAHP+ data (R2 = 0.4; Pearson’s correlation, *p < 0.001; n = 30 cells).

Two genetic markers have been identified in mice: transcriptional factor ER81/etv1 (sublayer 5a, slender apical dendrite) and glycosyltransferase-25, glt (sublayer 5b, thick apical dendrite), which allowed the identification of these neurons in acute experiments by linking their expression to fluorescent proteins (like GFP) in transgenic mice^5^. It allowed the adaptation patterns of ER81 and glt neurons to be linked to their morphology and the development of reliable protocols of neuronal identification^7^ (Fig. 1a,b), in turn revealing specific differences in ion channel expression^8^. Furthermore, ER81-positive neurons were shown to display a strong slow afterhyperpolarization (sAHP^7^).

sAHP was originally described in pyramidal cells as a slow, Ca^2+^-dependent afterpotential that controls spike frequency adaptation^9^. It has been well studied in hippocampal CA1 neurons. Recently, it has been demonstrated that potassium KCa3.1 channels mediate the sAHP in CA1 pyramidal cells using a selective blocker^10,11^. Another group found no evidence of potassium KCa3.1 channel contribution^12^, but this was subsequently refuted^11^. Later, the role of KCa3.1 channels in sAHP was confirmed^13^. KCa3.1 channels are activated by the calmodulin/Ca^2+^ complex and display intermediate conductance compared to SK (small potassium) and BK (big potassium) channels. The KCa3.1 gene, SK4 (KCNN4), displays ~ 40% homology with Ca-activated SK potassium channel genes^14^. Besides the hippocampus, KCa3.1 immunoreactivity was detected in neocortical L5 pyramidal cells^15^.

Although the sAHP of ER81+ neocortical pyramidal neurons and the sAHP of CA1 pyramidal neurons are similar and share some common features such as norepinephrine sensitivity^7,16^, which channels underlie the sAHP in L5 neocortical neurons remains unknown. Thus, the pharmacological properties of the unknown channels underlying the sAHP in L5 neurons, and how they are linked to the adaptation of spiking, an important characteristic of genetically and morphologically distinct neuronal subtypes in L5 remain to be investigated.

In the present work, we analyze the adaptation patterns of L5 neurons and how they are linked to the sAHP in a rodent model. We explore the sensitivity of the sAHP in strongly adapting L5 neurons to potassium channel blockers. Most importantly, we demonstrate the sensitivity of Ca^2+^-evoked currents L5 neurons with strong sAHP to a selective blocker of KCa3.1, as well as a co-localization of the ER81 and KCa3.1 immunoreactivity in L5 neurons.

## Results

Er81-positive neurons are known to display stronger adaptation than glt-positive neurons in the visual cortex, thus they can be referred to as adapting and nonadapting, respectively^5^. To measure the strength of adaptation of L5 cells, we recorded their spiking (~ 14–15 action potentials) induced by a continuous current step (duration: 1 s) and calculated the ratio of the last interspike interval to the third interspike interval (adaptation ratio; modified from^5^). To characterize the sAHP and correlate it with the adaption ratio, we induced 10 action potentials by a stimulation train at 50 Hz^7^ and measured the amplitude of afterhyperpolarization 300 ms after the end of the train (sAHP_300_). The amplitude of the train current was selected to evoke a single action potential with each pulse.

Typically, the cells with larger sAHP (sAHP+ cells; sAHP amplitude at 300 ms: 2.97 ± 0.15 mV; n = 30) displayed stronger adaptation (adaptation ratio: 2.63 ± 0.63, n = 30; Fig. 1a,b). Whereas the cells with residual sAHP (non-sAHP cells; sAHP amplitude at 300 ms: 0.13 ± 0.04 mV, n = 23) displayed weaker adaptation (adaptation ratio: 1.04 ± 0.015, n = 23; Fig. 1c,d). Next, we plotted the amplitudes of sAHP_300_ measured from the 50-Hz train protocols (Fig. 1e,f) against the adaptation ratios (Fig. 1g). The plot revealed two groups of cells distinguished by sAHP amplitudes with a clear gap in between. The sAHP + group displayed a significant correlation between sAHP amplitudes and adaptation ratios (Pearson’s correlation: R = 0.63, p < 0.001, n = 30).

**Figure 2:**
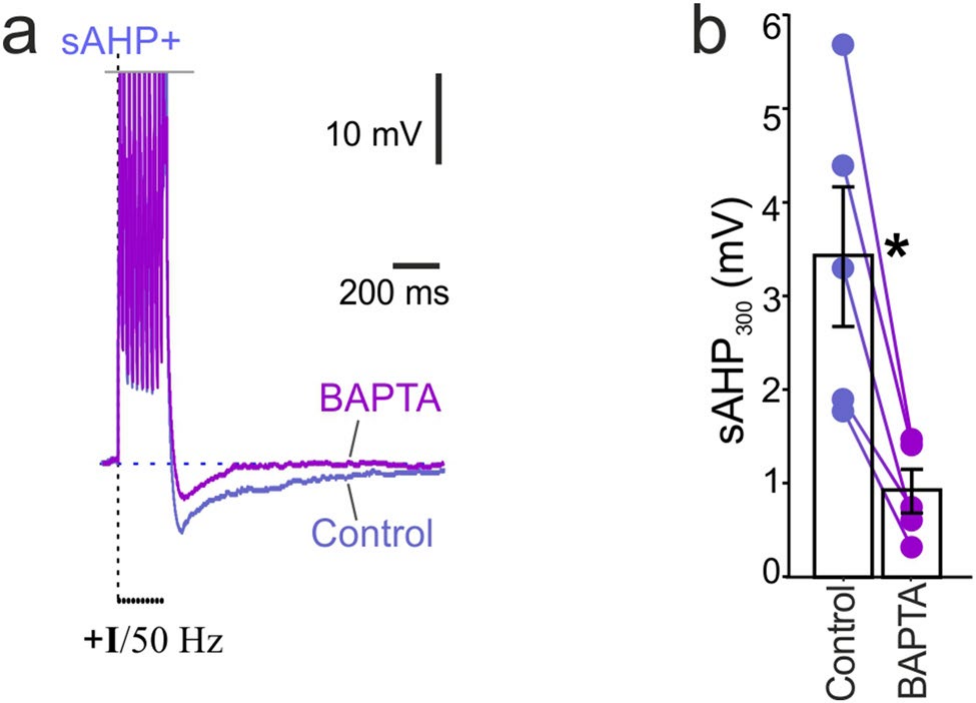
Intracellular application of BAPTA, a powerful chelator of Ca^2+^, blocked the sAHP. (**a**) Example traces of sAHP evoked by a 50-Hz train of action potentials after loading neurons with BAPTA vs. pretreatment control. (**b**) Summary diagram shows a significant difference between sAHP amplitudes after BAPTA loading compared to pretreatment control amplitudes (*paired t-test, n = 5, p < 0.05).

Previous studies have demonstrated that sAHP depends on calcium influx induced by APs^7,17^. In the next experiment, we elevated the cytoplasmic capacity of sAHP cells to buffer intracellular Ca^2+^ by loading the cells with BAPTA, a fast Ca^2+^ chelating agent (Fig. 2a). BAPTA loading resulted in a significant decrease of sAHP amplitudes at 300 ms compared to the pretreatment control measurements (control: 3.41 ± 0.76 mV, BAPTA: 0.90 ± 0.23 mV; paired t-test, t = − 5.53, n = 5, p < 0.01; Fig. 2b).

**Figure 3:**
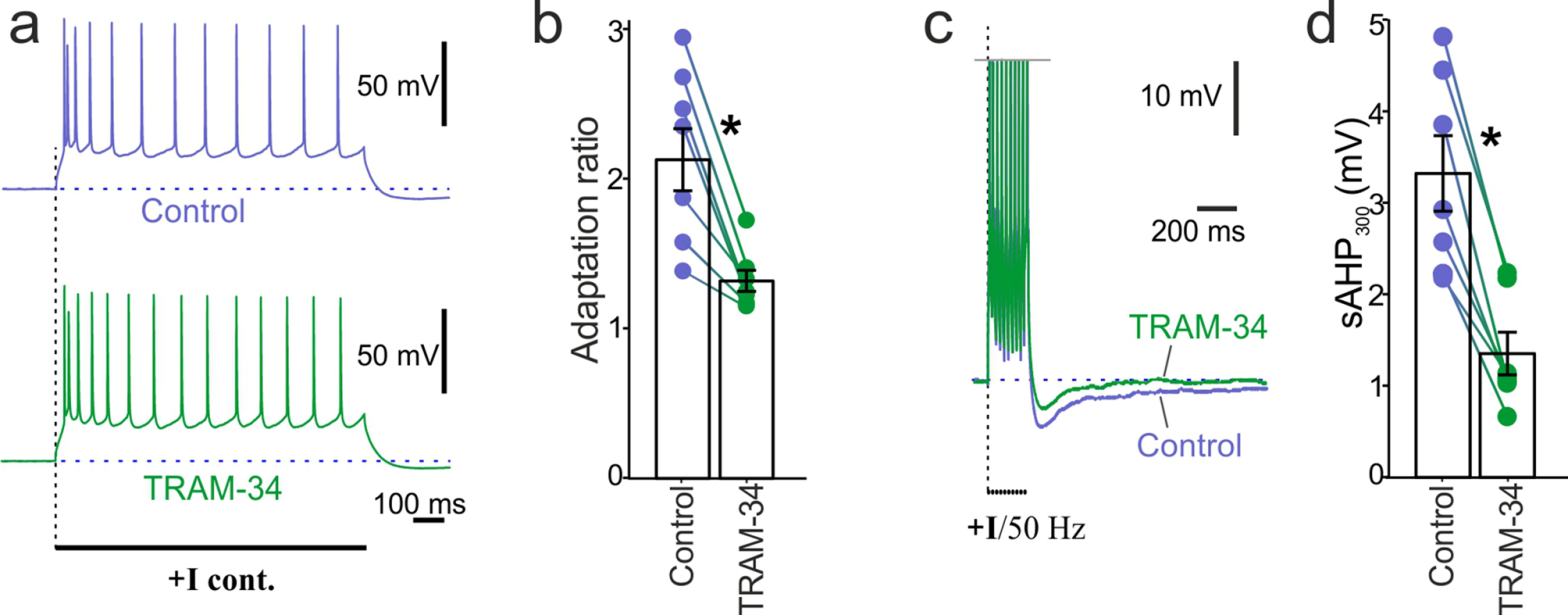
Bath application of TRAM-34, a powerful blocker of IK channels reduced sAHP and adaptation of evoked spiking. (**a**) Examples of adaptation patterns evoked by a 50-Hz train after application of TRAM-34 (5 µM) vs. pretreatment control recordings. (**b**) Summary diagram shows a significant decline of adaptation ratios after the application of TRAM-34 compared to pretreatment control amplitudes (*paired t-test, n = 7, p < 0.05). (**c**) Examples of sAHP evoked by a 50-Hz train of action potentials after application of TRAM-34 vs. pretreatment control. (**d**) Summary diagram shows a significant decrease of sAHP_300_ amplitudes after TRAM-34 application compared to pretreatment control amplitudes (*paired t-test, n = 7, p < 0.05).

In hippocampal pyramidal neurons, TRAM-34, a selective blocker of KCa3.1 channels is known to reduce the amplitude of sAHP^10,13^. Similarly, our experiments in sAHP+ cells of neocortical pyramidal layer 5 showed that TRAM-34 application (5 µM^13^) significantly reduced both the adaptation ratio (control: 2.13 + 0.21, TRAM-34: 1.32 ± 0.08; paired t-test, t = 5.07, n = 7, p < 0.01; Fig. 3a,b) and sAHP (control: 3.33 ± 0.42 mV, TRAM-34: 1.36 ± 0.24 mV; paired t-test, t = − 8, n = 7, p < 0.01; Fig. 3c,d) compared to the pretreatment control. To rule out the possibility of rundown of the sAHP^12^ we compared the effect of TRAM-34 application to the control recordings taken over the same period of time. The normalized decrease of the sAHP in TRAM-34-treated cells was stronger compared to rundown in control cells (TRAM-34-treated cells: 59.2 ± 7.4%, rundown control cells: 9.0 ± 2.8%; ANOVA, p < 0.05, n = 7, 7).

**Figure 4:**
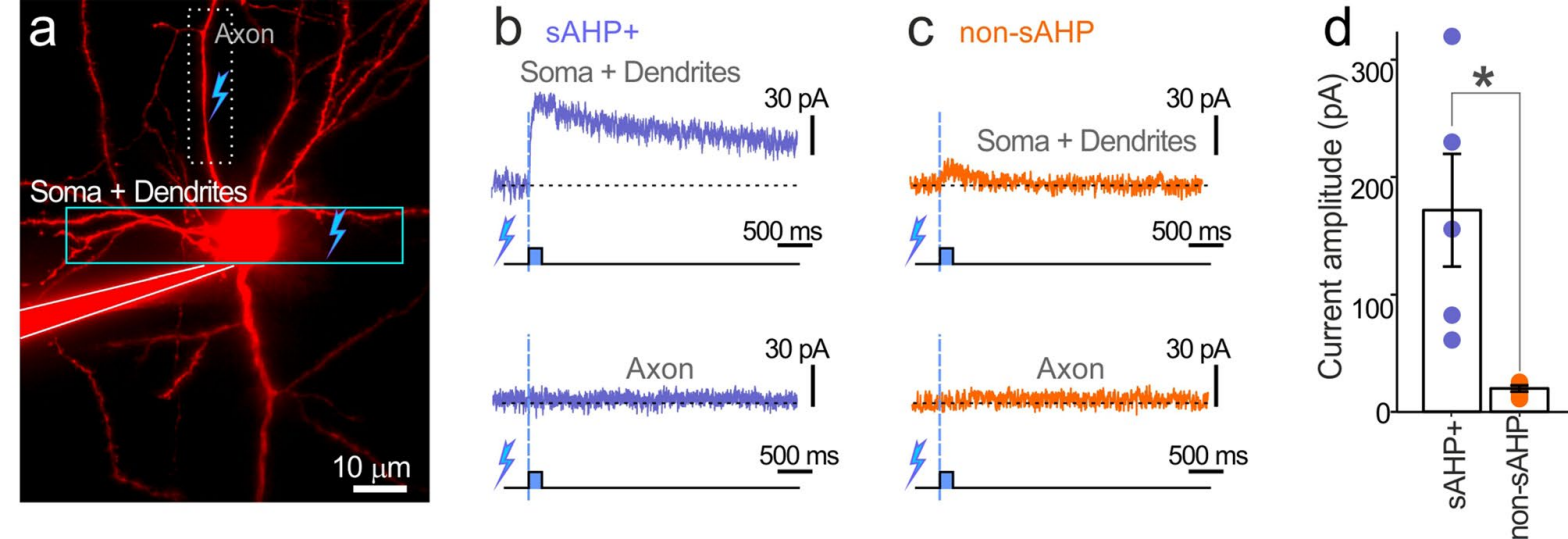
Local Ca^2+^ rise in the soma/dendrites but not in the axon induced hyperpolarizing current in voltage-clamped L5 cells. (**a**) Schematic for testing currents evoked by Ca^2+^ uncaging showing uncaging targets (squares). We identified axons as straight smooth (spineless) processes with the collaterals emerging at 100–120 mm from the soma. (**b**) Ca^2+^ uncaging in the soma and proximal dendrites of a sAHP+ cell induced a prolonged well-distinguishable hyperpolarizing current and no response when uncaging was applied to the proximal axon. (**c**) Ca^2+^ uncaging in the dendrites of a non-sAHP cell induced a marginal inward current and no response when uncaging was applied to the proximal axon. (**d**) The Summary diagram shows that amplitudes of induced currents were significantly larger in sAHP cells vs. non-sAHP cells (*ANOVA, n = 5, 5; p < 0.05).

To explore the possibility of KCa3.1 involvement in sAHP in more detail, we recorded currents induced by Ca^2+^ uncaging inside voltage-clamped neurons filled with caged Ca^2+^ through the patch pipette (Fig. 4a). Uncaging in the region of interest (ROI) encompassing the proximal axon induced no detectable currents in neither sAHP+ cells (n = 5; Fig. 4b) nor in non-sAHP cells (n = 5, Fig. 4c). Uncaging in the ROI of soma + proximal basal dendrites induced hyperpolarizing currents in both sAHP+ and non-sAHP cells (Fig. 4b,c). Overall, sAHP+ cells displayed significantly larger amplitudes of Ca^2+^-induced currents compared to non-sAHP cells (Fig. 4d; sAHP+ cells: 170 ± 50 pA; non-sAHP cells: 20 ± 3 pA; ANOVA, p < 0.05, n = 5, 5). Also, the sAHP+ cells displayed a slowly-deactivating component that was not pronounced in non-sAHP cells. These results allowed us to hypothesize that the late slowly deactivating current was related to the KCa3.1 channels and the sAHP.

**Figure 5:**
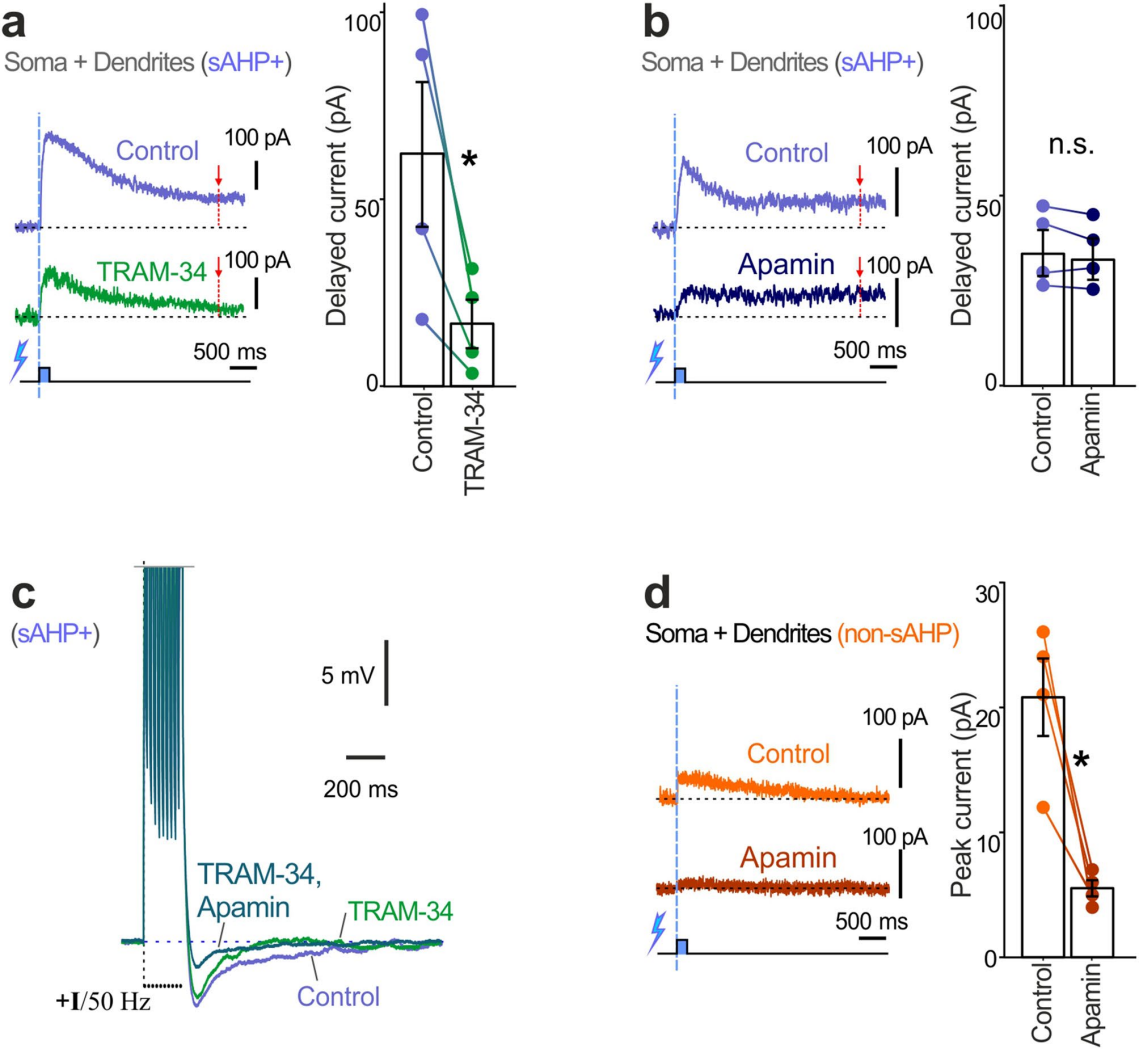
Rise of intracellular Ca^2+^ induced TRAM-34-sensitive outward current in sAHP+ cells. (**a**) Examples of currents evoked by Ca^2+^ uncaging in sAHP+ cells after bath application of TRAM-34 (5 µM) vs. pretreatment control. The summary diagram (right) shows a significant decrease of delayed current amplitudes (at 3 s after uncaging, red dotted lines and arrows) compared to pretreatment control amplitudes (*paired t-test, n = 4, p < 0.05). (**b**) Examples of currents evoked by Ca^2+^ uncaging in sAHP+ cells after bath application of apamin (100 nM) vs. pretreatment control. The summary diagram (right) shows no significant difference between amplitudes of delayed currents (at 3 s after uncaging, red dotted lines and arrows) compared to pretreatment control amplitudes (n.s. paired t-test, n = 4, P = 0.25). (**c**) Example of application of TRAM-34 (0.5 µM through the pipette) followed by subsequent bath application of apamin (100 nM) vs. pretreatment control. (**d**) Examples of currents evoked by Ca^2+^ uncaging in non-sAHP cells after application of apamin vs. pretreatment control. The summary diagram (right) shows a significant decrease in amplitudes of peak currents compared to pretreatment control amplitudes (*paired t-test, n = 4, p < 0.05).

Next, we applied TRAM-34, a selective blocker of KCa3.1 channels, and compared the amplitudes of late slowly deactivating currents evoked by Ca^2+^ uncaging in sAHP+ cells to the pretreatment control currents (Fig. 5a). To characterize the late current, we measured it at 3 s after flashing as the dynamics of uncaging-evoked Ca^2+^ elevation is much slower compared to that induced by action potentials^18^. In our experiments, TRAM-34 application to the bath significantly reduced the amplitudes of late currents (TRAM-34: 17 ± 6 pA, control: 63 ± 19 pA; paired t-test, t = 3.36, n = 4, p < 0.05). In the next series of reference experiments, the effect of apamin, an SK channel blocker, on the late current of sAHP+ cells was tested in sAHP+ cells. SK channels are common to both sAHP+ and non-sAHP cells, and blockade of SK channels diminishes medium afterhyperpolarization^7,19^. Like KCa3.1, the SK channel can be activated intracellularly by the CaM/Ca^2+^ complex. Thus, we applied apamin to dissect the current underlying the sAHP. In our experiments, apamin application did not reduce late currents in sAHP+ cells compared to pretreatment control (apamin: 32 ± 6 pA, control: 33 ± 6 pA; paired t-test, n = 4, t = 1.5, P = 0.25; Fig. 5b). TRAM-34 application followed by apamin application shows that KCa3.1 channels rather than SK channels contributed to the late slowly deactivating currents evoked by Ca^2+^ elevation (Fig. 5c). Adding to this, our experiments with apamin application to non-sAHP cells demonstrated that apamin greatly reduced the amplitudes of currents induced by Ca^2+^ uncaging in these cells (apamin: 20 ± 3 pA; control: 170 ± 50 nA, paired t-test, t = 4.88, n = 4, p < 0.05; Fig. 5d).

**Figure 6:**
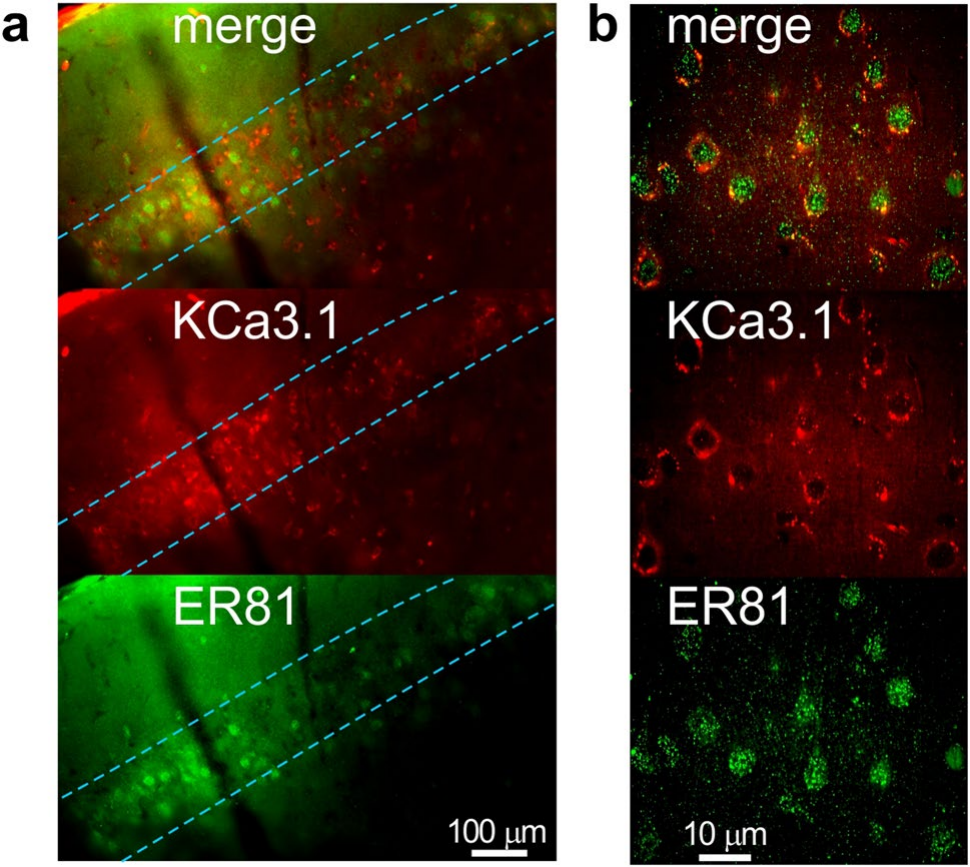
Immunohistochemical co-localization of ER81 (green), a marker of slender-tufted pyramidal cells in layer 5a with the KCa3.1 channel (red). (**a**) Conventional epifluorescent image of a double-labeled neocortical slice. Light blue dotted lines roughly demarcate the layer of ER81 expression. The top of the images is towards the surface of the neocortex. (**b**) Confocal image of an area inside the layer shown in “(**a**)” revealed co-localization of ER81 and KCa3.1 signals in individual cells.

Previous work has identified much larger sAHPs in ETV1 (ER81)-positive neurons with slender-tufted morphology located in sublayer 5A^7^. Thus, if KCa3.1 channels mediate the sAHP, they should co-localize with the genetic marker ER81 in the same cells. To test this hypothesis, we double-labeled neocortical slices with antibodies to ER81 and KCa3.1 (Fig. 6). Immunostaining experiments revealed a layer-like distribution of ER81 immunofluorescence in large pyramidal-like neurons (Fig. 6a). Within the layer, ER81 immunofluorescence (green) in the somas co-localized with KCa3.1 immunofluorescence in the somatic membranes (red, Fig. 6b). We analyzed 12 areas (200 ×  200 μm) within the putative layer of co-localization, where we observed 221 ER81-positive cells and 228 KCa3.1-positive cells in total (co-localization in the same cells: 94.4%).

**Figure 7:**
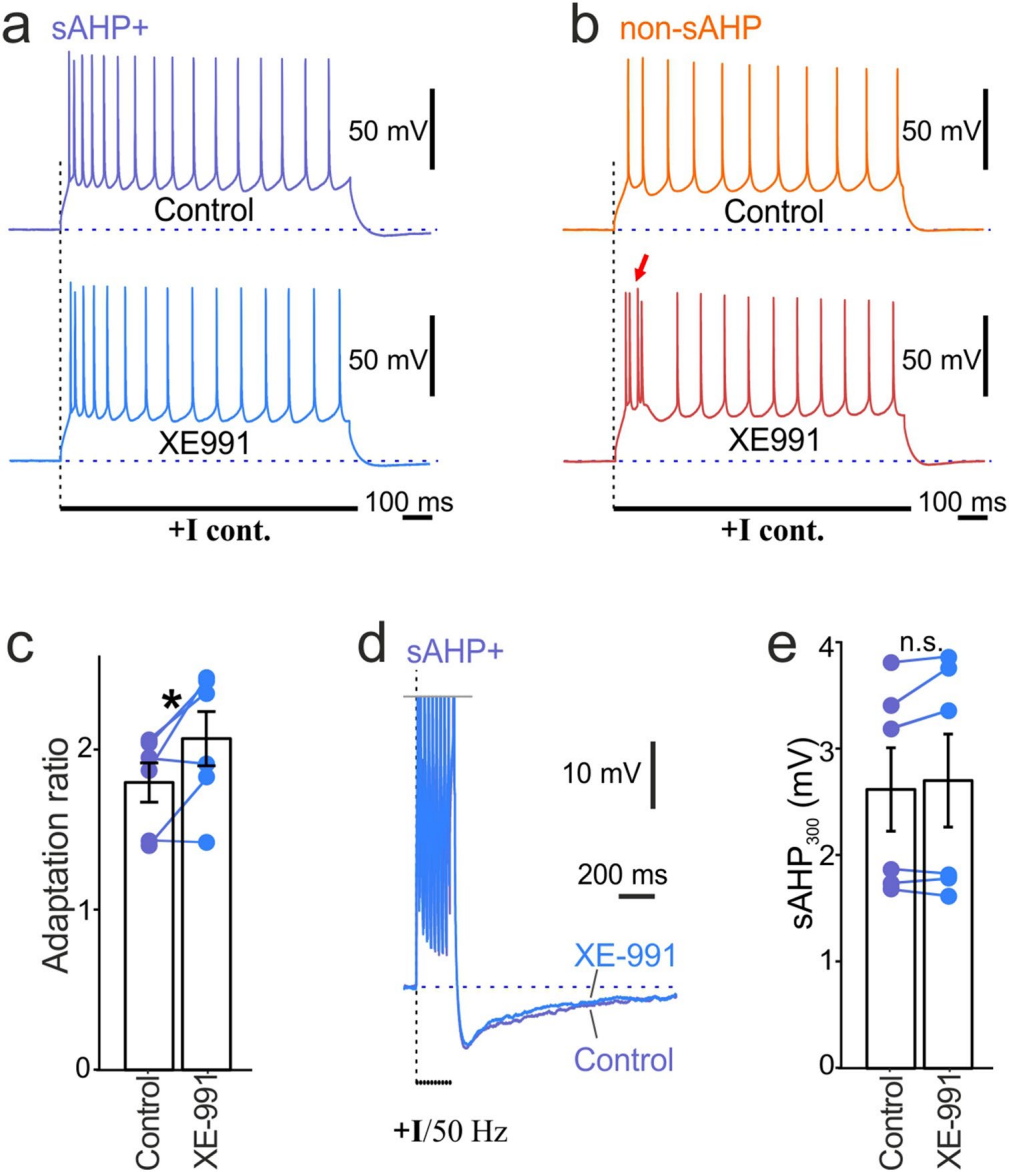
Bath application of XE-991, a blocker of Kv7 channels did not affect the sAHP. (**a**) Example of adaptation patterns of a sAHP+ cell with XE-991 wash in, compared to the pretreatment control stimulation. (**b**) Example of adaptation patterns of non-sAHP+ cell with XE-991 wash in compared to the pretreatment control stimulation. The arrow points at a burst of spikes. (**c**) The summary diagram shows a significant rise of adaptation ratios in sAHP+ cells after application of XE-991, compared to the pretreatment control coefficients (*paired t-test, n = 6, p < 0.05). (**d**) Examples of sAHPs evoked by a 50-Hz train of action potentials after application of XE-991 vs. pretreatment control. (**e**) Summary diagram shows no significant difference of sAHP_300_ amplitudes after XE-991 application compared to the pretreatment control amplitudes (n.s.: paired t-test, n = 6, P = 0.25).

Some pharmacological and genetic data suggest that Kv7 channels may mediate the sAHP^20,21^. However, in our uncaging experiments, we recorded no detectable Ca^2+^ mediated currents when we targeted the axon, a site of Kv7 channel expression in L5 pyramidal neurons^22^. To explore this possibility in more detail, we tested the effect of XE-991, a selective blocker of Kv7 channels on amplitudes of sAHP_300_ and adaptation ratios of sAHP+ cells (Fig. 7). Paradoxically, application of XE-991 increased adaptation in sAHP+ cells (XE-991: 2.01 ± 0.17; Control: 1.79 ± 0.13, paired t-test, t = 2.68, n = 6, p < 0.05; Fig. 7a,c). Unlike sAHP + cells, non-sAHP cells displayed bursting after XE-991 application (n = 4; Fig. 7b), which confirmed the previous observations^22^. No effect on the sAHP amplitude at 300 ms was detected using our sAHP-induction protocol (XE-991: 2.69 ± 0.44 mV; control: 2.6 ± 0.4 mV; paired t-test, t = − 1.3, n = 6, P = 0.25; Fig. 7d,e). XE-991 application induced similar normalized membrane depolarization in both sAHP + and non-sAHP cell groups (sAHP + cells: 5.6 ± 1.4% non-sAHP cells: 6 ± 1.1%, ANOVA, P = 0.55, n = 6, 5).

## Discussion

Here, we establish a relationship between the Ca^2+^-dependent sAHP and KCa3.1 channels in an intrinsic feedback mechanism that links activity in L5 pyramidal neurons to the adaptation of their spiking discharge. We show that the contribution of KCa3.1 channels is significant when the neuron fires a train of action potentials. To the best of our knowledge, our study is the first to link the KCa3.1 channels and the sAHP in neocortical neurons. Even more importantly, we demonstrate this link using several parallel methods: electrophysiology, pharmacology, immunohistochemistry, and photoactivatable probes. With immunohistochemistry, we identified the KCa3.1 cells as ER81-positive pyramidal neurons located in the neocortical layer 5. Targeted Ca^2+^ uncaging confirmed the two main features of KCa3.1 channels: preferential somatodendritic localization^15^ and Ca^2+^-driven gating. In addition, both the sAHP and the slow Ca^2+^-induced hyperpolarizing current were sensitive to TRAM-34, a blocker of KCa3.1 channels.

Our experiments reveal a strong similarity of the key features of KCa3.1 function in neocortical L5 neurons to those described in CA1 hippocampal neurons. Previous studies demonstrated that intermediate-conductance Ca^2+^-dependent KCa3.1 channels underlie the sAHP generated by trains of synaptic input or postsynaptic stimuli in CA1 hippocampal pyramidal cells^10^. The role of KCa3.1 channels in the sAHP has been independently confirmed^13^.

Compared to conventional electrophysiological studies, the uncaging of Ca^2+^ activates Ca^2+^-gated channels without the need for membrane depolarization^23^. Sah and Clements^24^ studied sAHPs in CA1 cells with Ca^2+^ uncaging using the same photosensitive component (DMNP-EDTA) we employed in this work. The shape of the Ca^2+^-evoked currents was generally similar to that recorded in our experiments from layer 5 neocortical sAHP+ cells. They called it the sAHP current, though they did not employ specific blockers to dissect the sAHP. Uncaging experiments^24^ and cell-attached patch-clamp experiments suggest that sAHP channels may be concentrated in the basal dendrites of CA1 pyramidal cells^25^.

Our experiments demonstrated SK- and KCa3.1-related conductances, which were blocked in sAHP+ cells by apamin and TRAM-34 independently. However, a recent study^26^ demonstrated that co-expression of SK1 and KCa3.1 subunits can produce heteromeric channels with intermediate properties, sensitive to TRAM-34 but without apamin sensitivity. Thus, the localization of KCa3.1 subunits in sAHP+ cells may affect the expression of their apamin-sensitive SK currents.

Kv7 potassium channels that underlie the “M current” were regarded as a candidate contributing to the sAHP^17^. However, the CaM/Ca^2+^ complex downregulates the neuronal isoforms (Kv7.2/7.3) of M-channel^27^. Our experiments with Ca^2+^ uncaging in the axons, the sites of Kv7 localization in L5 neurons^22^, demonstrated no Ca^2+^-evoked currents, whereas the same uncaging procedure evoked hyperpolarizing currents in the somato-dendritic compartment. KCa3.1 immunolabeling was localized primarily in the somatic region of excitatory cells in cortical structures^15^. Moreover, using channel blockers, Tivari et al.^13^ addressed the role of Kv7 channels in hippocampal CA1 pyramidal neurons and found no evidence for their contribution to the sAHP.

Apart from Ca^2+^-activated K^+^ channels, other contributors to the sAHP have been reported. Two groups emphasized an important role for a Na–K pump in producing the sAHP in hippocampal CA1 pyramidal cells^13,28^ and L5 neocortical pyramidal cells^28^. Moreover, a Slack/Slo co-assembly was strongly suggested as contributing to intermediate K^+^ conductance of the cortical neurons^29^.

In our work, the L5 cells fell into two distinct clusters: sAHP+ cells and non-sAHP cells. However, due to selection of the largest cells in the upper part of the layer, the L5 cells described in this work may not represent the whole population of layer 5.

ER81-expressing regular spiking L5 neurons with strong adaptation have been found in the barrel cortex^5^. These cells are distinguishable by the polarity of plasticity: they display deprived whisker depression but no spared whisker potentiation^30^.

## Methods

### Slice preparation

All experimental protocols were performed in accordance with the National Institutes of Health Guide for the Care and Use of Laboratory Animals and approved by the Bioethics Committee of the Institute of Higher Nervous Activity and Neurophysiology of Russian Academy of Sciences. Briefly, Wistar rats (P15-22) of both sexes were deeply anesthetized with isoflurane and decapitated. Brains were rapidly removed and placed in ice-cold artificial cerebrospinal fluid (ACSF). Frontal brain slices (350 μm) were cut using a vibratome (VT1200 S, Leica) from the primary visual cortex of the right hemisphere. The slicing angle was finely tuned to prevent damage to the apical dendrites of the surface-located L5 cells. ACSF contained 125 mM NaCl, 25 mM NaHCO_3_, 27.5 mM glucose, 2.5 mM KCl, 1.25 mM NaH_2_PO_4_, 2 mM CaCl_2_, and 1.5 mM MgCl_2_ (all Sigma BioXtra–graded; pH 7.4) and was aerated with 95% O_2_ and 5% CO_2_. The slices were incubated at room temperature for 90 min. Experiments commenced 90–120 min after slicing (for more detail see our previous work^18,31^).

### Electrophysiology

Patch pipettes (5 to 6 megaohms) were filled with intracellular patch solution: 132 mM K-gluconate, 20 mM KCl, 4 mM Mg–adenosine triphosphate, 0.3 mM Na2GTP, 10 mM Na-phosphocreatine, and 10 mM HEPES (pH 7.3) (all from Sigma). Brain slices were placed into a chamber continuously perfused with ACSF. Experiments were performed at or near physiological temperature (33 °C to 34 °C) and visualized under differential interference contrast (DIC) infrared optics (Axioskop 2 FS mot., Zeiss) with a Retiga Electro IR camera (QImaging). Membrane potential was recorded in whole-cell current clamp mode with an Axoclamp-2B amplifier and sampled at 20 to 50 kHz with a Digidata 1440A ADC (analog-to-digital converter) board using the Clampex 10 software (all from Molecular Devices (for more detail see our previous work^18,31^).

The adaptation ratio was calculated as average interspike time of the 14–15-th interspike interval (ISI) normalized to the average of the third ISI (i.e. excluding the 2 first ISIs). Thus, an adaptation ratio of 2 means that on average, the ISI interval increases ~ twofold after 10 spikes evoked by a continuous current step (Modified from Groh et al.^5^). Current amplitudes were selected to evoke ~ 14–15 spikes with a 1-s step. For our experiments, we selected the largest cells, which represent a significant portion of L5 pyramidal neurons, but not the whole population. Repetitively bursting cells were omitted and constituted ~ 1% of the cells recorded. To avoid a rundown of afterhyperpolarization due to compromised Ca^2+^ buffering capacity in weak cells, we omitted the cells that died or displayed a dramatic decrease in the amplitudes of their action potentials (> 10%) in the 10 min after the end of the experimental protocols.

We recorded currents in continuous voltage-clamp mode at a holding potential of − 55 mV, which is close to the calculated Nernst reversal potential of Cl^−^. The calculated reversal potential of K^−^ is  ~ − 110 mV, which guaranteed a substantial driving force through K^+^-permeable channels.

### Confocal imaging and uncaging

Live-cell imaging was performed with an LSM 5 LIVE DuoScan confocal microscope (Zeiss) equipped with a chromatically corrected water immersion lens (Plan Apochromat IR DIC 63×, 1.0 NA, Zeiss). Neurons were filled with the morphological tracer Alexa Fluor 594 hydrazide (100 μM, Invitrogen) and DMNP-EDTA (~ 5 mM; caged Ca^2+^, Biotium) by passive diffusion from the patch pipette for ~ 40 min. This caged compound is reliably decomposed inside neurons using a 405-nm laser without neuronal damage^32^. We imaged the silhouette of the cell using a 532-nm laser (50 mV) and 550 long-pass (LP) emission filter at ~ 1% of nominal laser power. We used the Alexa 594 signal to target relevant regions of the axon without inducing unwanted uncaging. A rectangular-shaped uncaging ROI was selected to encompass the target structure while minimizing the background region. The effectiveness of uncaging in increasing the local Ca^2+^ concentration was confirmed in our previous work in experiments that combined uncaging and Ca^2+^ imaging with a Ca^2+^ sensor^18^. In experiments with repetitive uncaging and blockers, we allowed 20–30 min after pretreatment control uncaging to reload the cell with DMNP-EDTA to restore its concentration before starting a second uncaging trial with a blocker in the bath. To ensure the matching of the uncaging region with the target structures established with Alexa 594 imaging, we projected the 405-nm uncaging laser beam using a chromatically corrected lens and the same scanner used for imaging. Confocal uncaging in voltage-clamped neurons allowed us to dissect Ca^2+^-activated transmembrane ionic currents from those activated by depolarization or hyperpolarization (for more detail see our previous work^18^).

### Pharmacology

We added blockers to the ASCF circulating in the perfusion system. TRAM-34 (Sigma) was dissolved in DMSO to 10 mM stock concentration (final: 5 µM). TRAM-34 inhibits the KCa3.1 channels and is ~ 1,000-fold more selective for KCa3.1 than the closely related SK channels^33^. We dissolved XE-991 (Sigma; stock: 100 mM, final: 10 µM) and apamin (Sigma; stock: 0.5 mM, final: 100 nM) in pure water. After each experiment with apamin application, we allowed ~ 7 days before starting the next one. In the experiments with induction of spiking, we employed additional blockers of synaptic transmission (CNQX (5 μM, Sigma), d-AP5 (20 μM, Tocris), and Picrotoxin (70 μM, Sigma) to avoid network-related effects. Cells were loaded with BAPTA (1 mM) mixed with the tracer Alexa Fluor 594 hydrazide by passive diffusion from the patch pipette for ~ 30 min. For intracellular application, we used 0.5 µM of TRAM-34 mixed with Alexa Fluor 594. The tip of the patch pipette was filled with clean intracellular solution for pre-loading control recording. The Kd of BAPTA and Ca^2+^ (~ 0.7 µM in the presence of Mg^2+^) is on the same order as the K_d_ of CaM and Ca^2+^^34^, which allowed BAPTA to compete with CaM for cytoplasmic Ca^2+^^23^.

### Immunohistochemistry

Deeply anaesthetized rats were perfused transcardially with phosphate-buffered saline (0.1 M PBS, pH 7.4) and 4% paraformaldehyde. Brains were removed and postfixed, rinsed in PB, then 50 µm sections were prepared on a VT1000s vibratome (Leica). All immunostaining procedures were performed on free-floating sections. Prior to incubation with the primary antibody solution, sections were washed for 2 h in a blocking solution. The blocking solution contained 0.5% Triton X-100, 0.01% sodium azide, 2% normal goat serum (Sigma), and 1% BSA (Sigma) in PBS. The primary antibodies, used in parallel, were anti-ER81 antibody raised in rabbit (840401, BioLegend, 1:10,000), and IK1 (KCa3.1) antibody (sc-365265, Santa-Cruz, 1:100), raised in mouse. Time of incubation in the primary antibody mixture was 72 h at 40 °C. Secondary antibodies, used simultaneously, were Alexa-488-conjugated goat anti-rabbit IgG (1:100, Molecular Probes) and Alexa-594-conjugated goat anti-mouse IgG (1:100, Molecular Probes). Time of incubation in the secondary antibody mixture was 72 h at 40 °C. In the control experiments, the primary antiserum was omitted. No staining was observed in this series. Washed with PB, sections were embedded in Aqua Poly/Mount (Polysciences, Inc) and studied using a 4070 M-GE digital camera (Thorlabs, USA) attached to an Axioskop-2 FS plus microscope (Zeiss, Germany) equipped with a chromatically corrected lens (Plan Apochromat 20×, 0.8 NA, Zeiss). Confocal imaging was performed using a chromatically corrected oil immersion lens (Plan Apochromat 63×, 1.4 NA, Zeiss).
